# Management of peripheral arterial disease in diabetes: a national survey of podiatry practice in the United Kingdom

**DOI:** 10.1186/s13047-018-0270-5

**Published:** 2018-06-08

**Authors:** Pasha Normahani, Chira Mustafa, Nigel J. Standfield, Claire Duguid, Martin Fox, Usman Jaffer

**Affiliations:** 1 Department of Vascular Surgery, Imperial College NHS Healthcare Trust, London, UK; 2 Department of Medicine, Royal Berkshire Hospital, Reading, UK; 3 Department of Podiatry, University of East London, London, UK; 4 Department of Podiatry, Pennine Acute Hospitals Trust, Manchester, UK; 5 Department of Vascular Surgery, Hammersmith Hospital, Du Cane Road, London, W12 0HS UK

**Keywords:** Macrovascular disease, Diabetic foot, Health care delivery

## Abstract

**Background:**

We aimed to investigate podiatry practice in diagnosing peripheral arterial disease (PAD) in diabetes, decision making once PAD is suspected and limitations of referral pathways.

**Methods:**

A survey, comprising 26 questions was distributed to podiatrists across the UK via mailing lists of collaborating organizations including the College of Podiatry (UK). Response rates were estimated based on NHS workforce data. Analysis of responses from the open-ended questions was performed using inductive content analysis.

**Results:**

Data from 283 respondents were analyzed. Response rate for all NHS podiatrists across the UK was estimated to be 6%. For the detection of arterial disease only 18.8% (*n* = 49/260) of participants reported using a full combination of history, pulse palpation, Doppler and ABPI assessment. Self-reported confidence in detecting arterial disease was highest amongst podiatrists who felt they had received adequate training compared to podiatrists who felt they had not (median 85 (IQR 75–90) vs 67 (50–77), respectively; *p* < 0.001) as well as those who see > 20 diabetic patients per week compared to those who see < 20 (median 80 (IQR 70–90) vs 72 (60–82.8), respectively; *p* < 0.001). Over one third of respondents (35.8%, *n* = 93/260) were aware of missed cases of PAD in the past year and 17.5% (*n* = 38/217) believed that this resulted in an amputation in some cases.

The survey highlighted a lack of clarity amongst podiatrists regarding referral guidelines. Additionally, 69% (*n* = 169/242) reported that their patients had to wait longer than 2-weeks for specialist vascular assessment and 67.6% (*n* = 54/80) reported similar waits for a Duplex Ultrasound scan. There was a statistically significant variation in DUS waiting time across the UK (X^2^ (10, *N* = 80) = 21.59, *p* = 0.017). Inability to make a direct referral to vascular services and long delays were reported as major limitations of the referral pathway.

**Conclusion:**

We have identified important targets for further investigation and quality improvement.

**Electronic supplementary material:**

The online version of this article (10.1186/s13047-018-0270-5) contains supplementary material, which is available to authorized users.

## Background

Prevention and management of diabetic foot ulceration requires complex, well-coordinated multidisciplinary care across all healthcare settings as recommended by the National Institute of Health and Care Excellence (NICE) and the ‘Putting Feet First’ National Framework [1, 2]. This care pathway is structured to include a ‘Foot Protection Team (FPT)’ to work in the community, comprising healthcare professionals, often podiatrists, with specialist expertise in diabetic foot assessment and management. The FPT work closely with a ‘Multidisciplinary Foot Care Team (MDFT)’ who manage diabetic foot problems in hospitals as well as more complex cases in the community.

Regular assessment of vascular status by the FPT is of utmost importance in the management of the diabetic foot to detect the presence of peripheral arterial disease (PAD). PAD is a major risk factor for the development of ulceration, amputation and overall mortality [3]. The detection of PAD in the non-ulcerated foot allows for correct identification of risk status and ulcer prevention. Furthermore, it provides an important opportunity to modify cardiovascular risk factors, which may impact positively on mortality rates [4]. Moreover, the prompt and accurate detection of PAD in the ulcerated foot is even more time critical as time to revascularisation is a determinant of ulcer healing and limb salvage [5, 6]. With late diagnosis of PAD, consideration of revascularisation is delayed and hence often unsuitable due to progression of tissue loss or infection [7–10]. Reported delays to vascular assessment and intervention in the literature negatively affect patient outcomes [11, 12].

Time to revascualrisation is potentially influenced by several variables including factors related to the patient (e.g. delay to presentation), health provider (e.g. delay in diagnosis, deficiency in knowledge and training) and health care system (e.g. referral pathways, waiting lists, staffing). Current national data sets from England, provide very little information regarding practice patterns in PAD diagnosis, decision making once PAD is suspected and the status of vascular surgery referral pathways [13]. In this national survey of podiatry practice in the United Kingdom (UK), we aimed to investigate current practice in diagnosing PAD in diabetes, decision making once PAD is suspected and limitations of referral pathways. This may help identify potential targets for further investigation and quality improvement. We have focused specifically on podiatry practice, as podiatry practice is the link between diabetic patients and diabetic foot care.

## Methods

A survey comprising of 26 questions was drawn up (Additional file 1: Figure S1). Questions were generated following literature review and discussion with a collaborative faculty including a podiatrist, podiatric surgeon and two vascular surgeons within our network. Following completion of the survey one question (“which patients do you routinely screen for peripheral arterial disease (PAD)?”) was removed as it was felt to be vague.

The remaining 25 questions were split into four categories: Demographics (7 questions); PAD assessment (7 questions); Vascular referrals (11 questions) (Additional file 1: Figure S1). Using online survey software (https://www.surveymonkey.co.uk) a link to questions was sent to mailing lists of the College of Podiatry (UK), alumni networks of major podiatry schools in the UK and local podiatry networks as well as relevant social media contacts of the authors between July to September 2016. A reminder email was sent two months following the first email to improve the response rate.

### Estimating response rates

As many of the survey invitations were sent by collaborating organisations, response rate could not be formally established or estimated based on how many podiatrists were in each group as this information was not always available. Therefore, we used NHS workforce statistics to estimate response rates and determine how representative the respondents were in terms of geographical spread. In order to establish the number of podiatrists in post across the UK, a number of reports were used; NHS England (July 2016) [14, 15], NHS Scotland (June 2016) [16], NHS Wales (September 2016) [17] and NHS Northern Ireland (March 2016) [18]. Detailed information regarding podiatrist demographics (banding) was only available from NHS England data, and so this was used to determine how representative the respondents were in terms of experience. Response rates for podiatrists working in the private sector could not be estimated due to a lack of available data regarding the size of the workforce.

### Statistical and qualitative analysis

Responses to the survey were downloaded and analyzed using Excel (Microsoft, Redmond, USA) and SPSS version 23 (IBM, New York). Continuous data demonstrated a non-normal distribution. Mann-Whitney U test was used to compare groups of continuous data. Categorical data were compared using Chi-squared test. Cut offs used for group comparisons (e.g. comparisons made between podiatrists seeing < 20 patients per week as compared to those seeing > 20 per week) were predetermined based on what was felt to be clinically relevant. A *p*-value of < 0.05 was considered statistically significant.

Analysis of responses from the open-ended questions was performed using inductive content analysis [19]. This involved reading all responses (author PN) and freely generating categories to describe all aspects of the content. Categories were named using content characteristic words and these were expanded into subcategories with similar themes. Using this abstraction method, a general description of responses could be created.

## Results

### Demographics

There were two hundred and eighty six respondents. Three non-podiatrist respondents were removed from the survey (two Vascular Nurse Specialists and a Vascular Scientist). The remaining data from 283 respondents were analyzed. Demographic data of respondents are presented in Table 1. Not all participants completed all of the questions in the survey. Therefore, the total number of respondents for each question is given in brackets (‘n=’) and percentages calculated using this denominator.

**Table 1:** Demographics

Occupation	(*n* = 283)
Podiatrist	274 (96.8%)
Podiatry consultant	7 (2.5%)
Podiatry assistant	2 (0.7%)
How many years have you worked in this capacity?	(*n* = 283)
< 5 years	71 (25.1%)
5–10 years	50 (17.7%)
> 10 years	162 (57.2%)
Which sector do you work in?	(*n* = 281)
NHS	239 (85.1%)
Private	42 (14.9%)
What is your banding?	(*n* = 236)
Band 4	1 (0.4%)
Band 5	31 (13.1%)
Band 6	89 (37.7%)
Band 7	86 (36.4%)
Band 8	27 (11.4%)
Band 9	2 (0.8%)
In which region of the UK do you work?	(*n* = 283)
East	11 (3.9%)
East Midlands	8 (2.8%)
London	39 (13.8%)
North East	3 (1.1%)
North West	42 (14.8%)
Northern Ireland	4 (1.4%)
Scotland	45 (15.9%)
South East	51 (18%)
South West	21 (7.4%)
Wales	23 (8.1%)
West Midlands	8 (2.8%)
Yorkshire and the Humber	28 (9.9%)
How many diabetic patients do you see per week?	(*n* = 283)
None	4 (1.4%)
< 5	22 (7.8%)
5–10	30 (10.6%)
10–20	45 (15.9%)
> 20	182 (64.3%)
How long is your typical appointment slot?	(*n* = 283)
0–10 min	2 (0.7%)
10-20 min	59 (20.9%)
20–30 min	176 (62.2%)
> 30 min	46 (16.3%)

We estimate that the response rate from NHS podiatrists across the country was approximately 6%. This is based on an estimated number of 3791 podiatrists working in the NHS across the UK around the time of the survey and the 239 NHS podiatrist respondents.

When comparing the geographical spread of respondents to the spread of podiatrists across the UK, we note an underrepresentation of the North East (1.1% vs 5.7%, respectively), West Midlands (2.5% vs 8.1%) and Northern Ireland (1.4% vs 5.6%) and an over representation of London (13.8% vs 9.6%) and the South East (18% vs 6.7%) (Additional file 2: Table S1).

We also compared the distribution of the respondents experience to the distribution across England and found an over representation of band 6–7 podiatrists (74.2% vs 42.9%, respectively) as well as an under representation of band 4–5 (14.4% vs 20.7%) and band 8–9 podiatrists (20.8% vs 36.3%).

### Assessment for arterial disease in diabetes

Podiatrists were confident in their ability to detect PAD in diabetes with a mean self-assessed confidence score of 76 (SD ± 16.8, *n* = 260) on a scale of 0 (least confident) to 100 (most confident). Podiatrists who see more than 20 patients per week were more confident in their ability to detected PAD compared to those who see less than 20 patients per week (median 80 (IQR 70–90) vs 72 (60–82.8), respectively; *p* < 0.001). Most felt that they had received adequate training in PAD detection (Yes, 63.5%; No, 23.5%; Unsure, 13.1%. *n* = 260). Those who felt they had received adequate training were more confident in their ability to detect PAD compared to those who felt they had not (median 85 (IQR 75–90) vs 67 (50–77), respectively; *p* < 0.001).

**Fig. 1:**
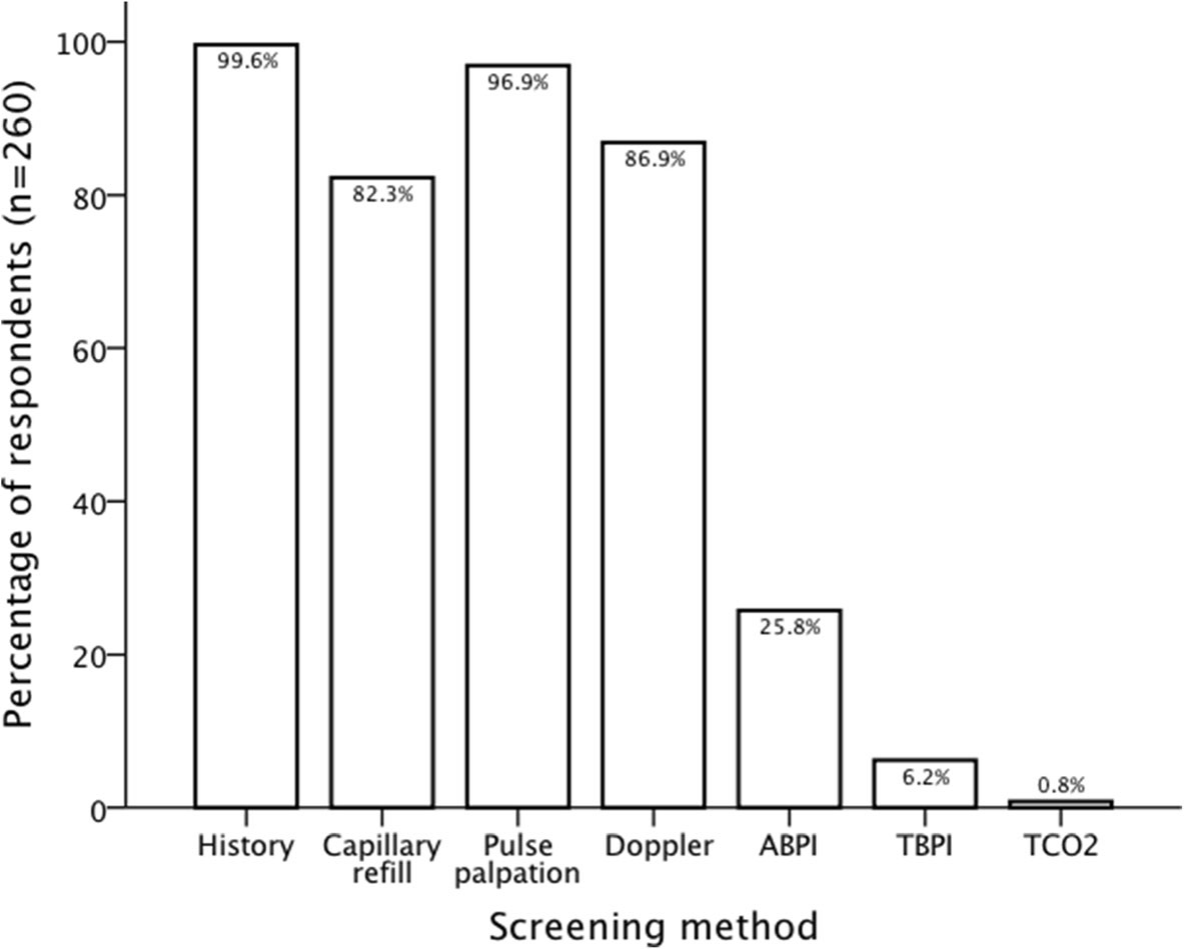
Bar chart representing responses to question “Which of the following do you routinely perform as part of your screening (tick all that apply) for PAD?”. Doppler: audible hand held Doppler waveform assessment; ABPI: ankle brachial pressure index; TBPI: toe brachial pressure index; TcPO_2_: transcutaneous oxygen pressure

**Fig. 2:**
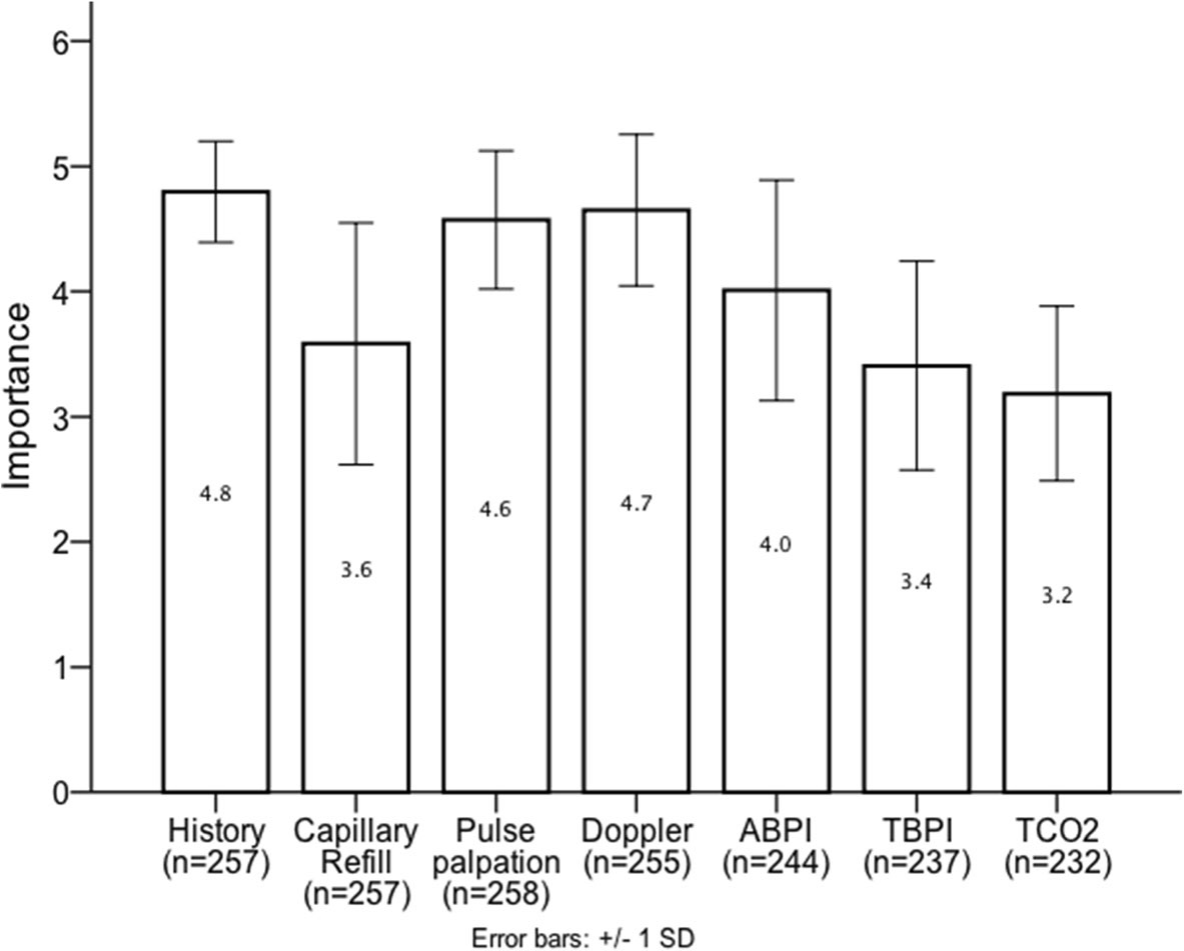
Bar chart representing responses to question “Please score the following options to indicate their importance to your clinical decision making.” Doppler: Audible hand held Doppler waveform assessment; ABPI: Ankle brachial pressure index; TBPI: Toe brachial pressure index; TcPO_2_: Transcutaneous oxygen pressure. SD(Standard deviation). Scored on a scale of 0 to 5; 0: not at all important; 1: not important; 2: unsure; 3: important; 5: very important

Respondents were asked regarding routinely performed PAD screening tests used in clinical practice and their relative importance in clinical decision-making (*on a scale of 1 to 5*: 1, not at all important; 2, not important; 3, unsure; 4, important; 5, very important). Their responses are presented in Figs. 1 and 2. Toe brachial pressure index (TBPI) and Transcutaneous pressure of oxygen (TcPO_2_) were seldom used in clinical practice (6.2 and 0.8% respectively) and were also scored as least important in clinical decision-making (3.4 and 3.2 respectively). A minimum combination of history and pulse palpation was used for PAD assessment by 96.2% of respondents (*n* = 260). In addition to history and pulse palpation at least one other non-invasive test was also used by 85.8% (*n* = 260). As the demonstrated in Table 2, the most common combination of tests, used by 60% of all respondents, was that of history, pulse palpation and audible Doppler waveform assessment. The second most common combination, used by 18.8% of all respondents, was that of history, pulse palpation, Doppler assessment and ABPI. There was a statistically significant association between region of work and the various combinations of tests used (X^2^ (121, *N* = 260) = 153.36, *p* = 0.025) (Table 2), with more than a third of respondents from East of England, North East and West Midlands using history, pulse palpation and at least two other non-invasive tests. There was no significant association between combination of tests used and the number of patients seen per week (X^2^ (11, *N* = 260) = 8.27, *p* = 0.69) or the perception of training adequacy (X^2^ (22, *N* = 260) = 13.69, *p* = 0.91).

**Table 2:** Combination of methods used to detect arterial disease by survey respondents according to region of work. Data represent the percentage count (%) for each region (row)

	Combination of methods												
Region	Hx (*n* = 1)	Hx + pulse (*n* = 31)	Hx + Doppler (*n* = 5)	Hx + pulse + ABPI (*n* = 1)	Hx + pulse + Doppler (*n* = 156)	Hx + Doppler + ABPI (*n* = 1)	Hx + pulse + ABPI + Doppler (*n* = 49)	Hx + pulse + Doppler + TBPI (*n* = 1)	Hx + Doppler + ABPI +TBPI (*n* = 1)	Hx + pulse + Doppler + ABPI+TBPI	Hx + pulse + ABPI + TBPI + TCO2	Hx + pulse + Doppler + ABPI + TBPI + TCO2	TOTAL (%)
East (*n* = 11)	0	18.2	0	0	27.3	0	54.5	0	0	0	0	0	100%
East Midlands (*n* = 7)	14.3	0	0	0	71.4	0	14.3	0	0	0	0	0	100%
London (*n* = 33)	0	3	12.1	0	66.7	0	9.1	3	0	6.1	0	0	100%
North East (*n* = 3)	0	0	0	0	66.7	0	33.3	0	0	0	0	0	100%
North West (*n* = 40)	0	7.5	2.5	0	62.5	0	10	0	0	17.5	0	0	100%
Northern Ireland (*n* = 4)	0	25	0	0	50	0	25	0	0	0	0	0	100%
Scotland (*n* = 44)	0	18.2	0	0	61.4	0	18.2	0	0	2.3	0	0	100%
South East (*n* = 42)	0	9.5	0	0	61.9	0	23.8	0	0	0	2.4	2.4	100%
South West (*n* = 21)	0	14.3	0	0	71.4	0	9.5	0	0	4.8	0	0	100%
Wales (*n* = 21)	0	14.3	0	4.8	57.1	4.8	14.3	0	0	4.8	0	0	100%
West Midlands (*n* = 8)	0	12.5	0	0	50	0	37.5	0	0	0	0	0	100%
Yorkshire (*n* = 26)	0	19.2	0	0	50	0	26.9	0	3.8%	0	0	0	100%
TOTAL (%) (*n* = 260)	0.4%	11.9%	1.9%	0.4%	60%	0.4%	18.8%	0.4%	0.4%	4.6%	0.4%	0.4%	100%

*Hx* history, *Pulse* pulse palpation, *Doppler* audible hand held Doppler waveform assessment, *ABPI* ankle brachial pressure index, *TBPI* toe brachial pressure index, *TcPO*_*2*_ transcutaneous oxygen pressure

When asked whether ABPI (ABPI; < 0.9 considered abnormal) is a reliable method to exclude PAD in diabetes, most respondents felt that it was not (Yes 20%, No 57.7%, Unsure 22.3%; *n* = 260).

When podiatrists were asked whether in the past year they were aware of patients with diabetes who were diagnosed with PAD that had been previously missed, over a third of respondents reported that they were aware of such cases (Yes 35.8%, No 35.8%, Unsure 28.5%; *n* = 260). There was no statistically significant association between the perception of missed PAD detection and the region of work (X^2^ (22, *N* = 260) = 21.70, *p* = 0.48) or the combination of tests used in PAD screening (X^2^ (22, *N* = 260) = 18.65, *p* = 0.67). We went on to ask whether delayed detection of PAD in any of these cases had led to a minor or major amputation, to which 17.5% responded that it had, 33.8% responded that it had not and 49.3% were unsure (*n* = 217).

### Vascular referral pathways

When podiatrists were asked if they had access to advice from a vascular surgeon via a MDFT, a large proportion responded that they did not (Yes 59.4%, No 36.4%, Unsure 4.4%; *n* = 283). There was no association between access to vascular advice and region of work (X^2^ (22, *N* = 283) = 28.2, *p* = 0.17). However, there was a statistically significant association between access to vascular advice and the number of patients seen per week (X^2^ (2, *N* = 283) = 26.50, *p* < 0.001); 70% of podiatrists who see more than 20 patients per week reported having access to vascular advise as compared to 40% of podiatrists who see less than 20 patients per week.

**Fig. 3:**
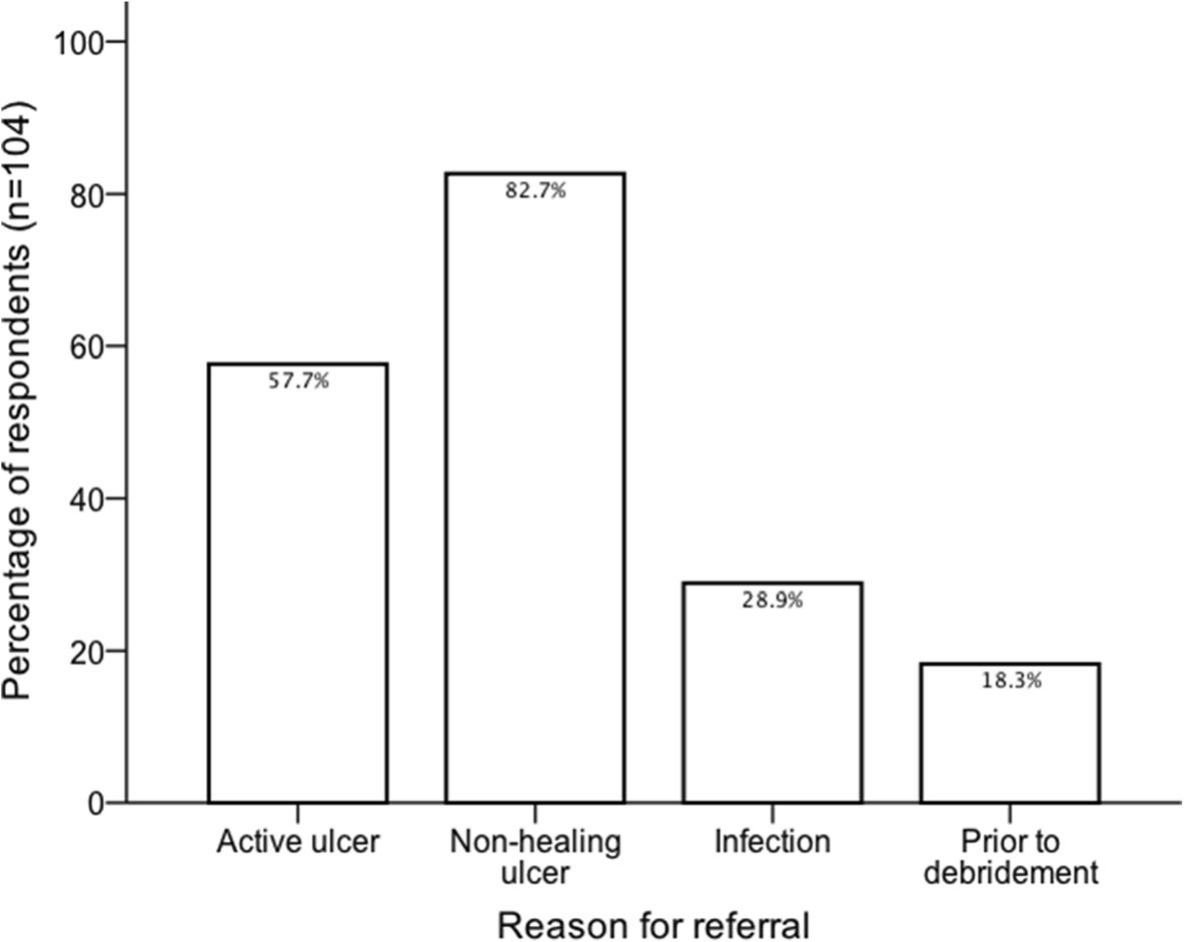
Bar chart representing responses to question “If you suspect PAD in a patient with diabetes, when would you refer for further Vascular assessment?” (multiple choice). Respondents who reported that they would refer ‘any patient with suspected PAD and diabetes for further assessment’ have been excluded from this graph. Active ulcer; if there is any active foot ulceration. Non-healing ulcer; if there is an ulcer that has not improved within 6-weeks despite optimal management. Infection; if there is evidence of infection. Prior to debridement; if they need local debridement

In a multiple-choice question, most respondents reported that they would refer ‘any patient with suspected PAD and diabetes for further assessment’ (59.4%, *n* = 256). Amongst the remaining respondents (*n* = 104) the most common triggers for vascular referral were active ulcers and non-healing ulcers (Fig. 3). On further analysis, 43 (16.8%) respondents reported that they would only refer patients with PAD who had non-healing ulcers despite 6-weeks of optimal management.

In terms of volume of referrals, respondents reported on average referring six patients (SD ±16.5, *n* = 225) per month or 41 patients (SD ± 63.4) per year (*n* = 207).

**Fig. 4:**
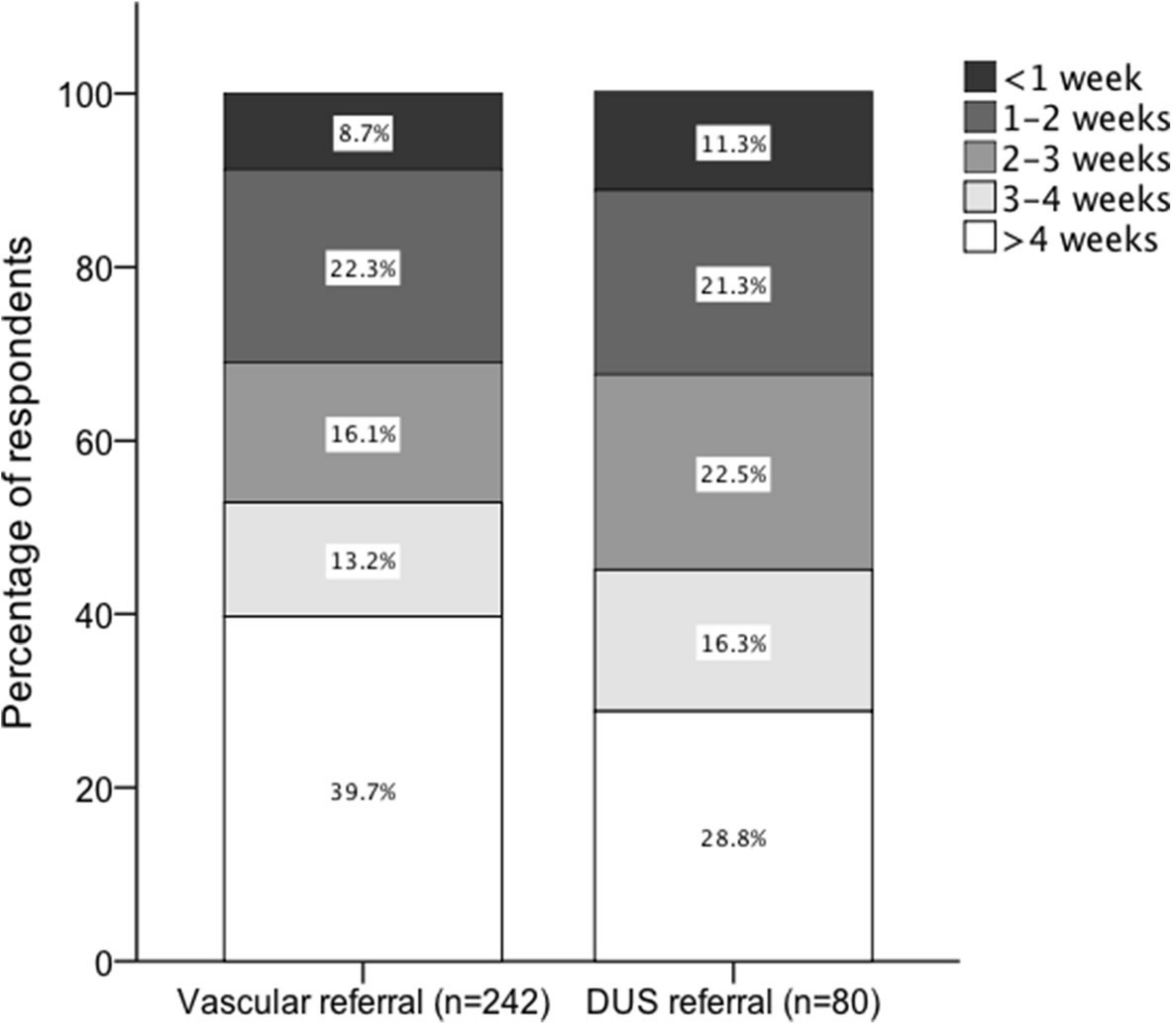
Stacked bar chart representing responses to the following two questions: “on average how long do your patients have to wait for a Vascular assessment by a vascular surgeon?” and a follow on question for those who can directly request a duplex ultrasound (DUS) “if yes, how long does it take to have a duplex ultrasound performed?”

The reported waiting times for vascular review are presented in Fig. 4. Most respondents felt that waiting times for vascular assessment were too long (appropriate waiting time 28.1%, too long 50.4%, unsure 21.5%; *n* = 242). There was no statistically significant association between reported vascular waiting times (groups compared: > 4 weeks or < 4 weeks) and the number of patients seen per week (groups compared: > 20 patients per week or < 20 patients per week) (X^2^ (1, *N* = 242) = 1.05, *p* = 0.19) or with the region of work (X^2^ (11, *N* = 242) = 17.33, *p* = 0.10).

When asked whether they always receive the outcome of the vascular consultation, the majority reported that they do not (yes 28.1%, no 71.9%; *n* = 256). This was not associated with the number of patients seen per week (X^2^ (1, *N* = 256) = 0.53, *p* = 0.28) or the region of work (X^2^ (11, *N* = 256) = 14.95, *p* = 0.19).

When asked what proportion of patients referred to Vascular Surgery over the past year required vascular intervention, responses were mixed: *< 10,* 19.4%; *10–25*, 16.1%; *25–50*, 19%; *50–75*, 26%; *> 75*, 19.4%.

Most of those surveyed reported that they were unable to request a Duplex Ultrasound (DUS) directly (Yes 20.6%, no 69.4%, unsure 9.9%; *n* = 242). The average waiting times, for those who could, are presented in Fig. 4. Most respondents felt that the waiting times were too long (appropriate waiting time 26.6%, too long 42.6%, unsure 30.9%; *n* = 90). There was no statistically significant association between the ability to request a DUS and number of patients seen per week (X^2^ (2, *N* = 242) = 4.11, *p* = 0.13) or the region of work (X^2^ (22, *N* = 242) = 30.21, *p* = 0.11). However, there was a statistically significant association between duration of wait (> 4 weeks or < 4 weeks) and the region of work (X^2^ (10, *N* = 80) = 21.59, *p* = 0.017). A large number of respondents reported waiting times of less than 4 weeks in London (84.6% reported waiting times of < 4 weeks, *n* = 13), North West (85.7% < 4 weeks, *n* = 14), South East (91.7% < 4 weeks, *n* = 12), North East (100% < 4 weeks, *n* = 2), East Midlands (100% < 4 weeks, *N* = 1), West Midlands (100% < 4 weeks, *n* = 1) and Yorkshire (87.5% < 4 weeks, *n* = 8). Conversely, a larger proportion of respondents reported waiting times > 4 weeks in East (66.7% waited > 4 weeks, *n* = 3), South West (66.7% > 4 weeks, *n* = 6) and Wales (63.6% > 4 weeks, *n* = 11). There was a mixed response from Scotland (55.6% < 4 weeks, *n* = 9).

**Fig. 5:**
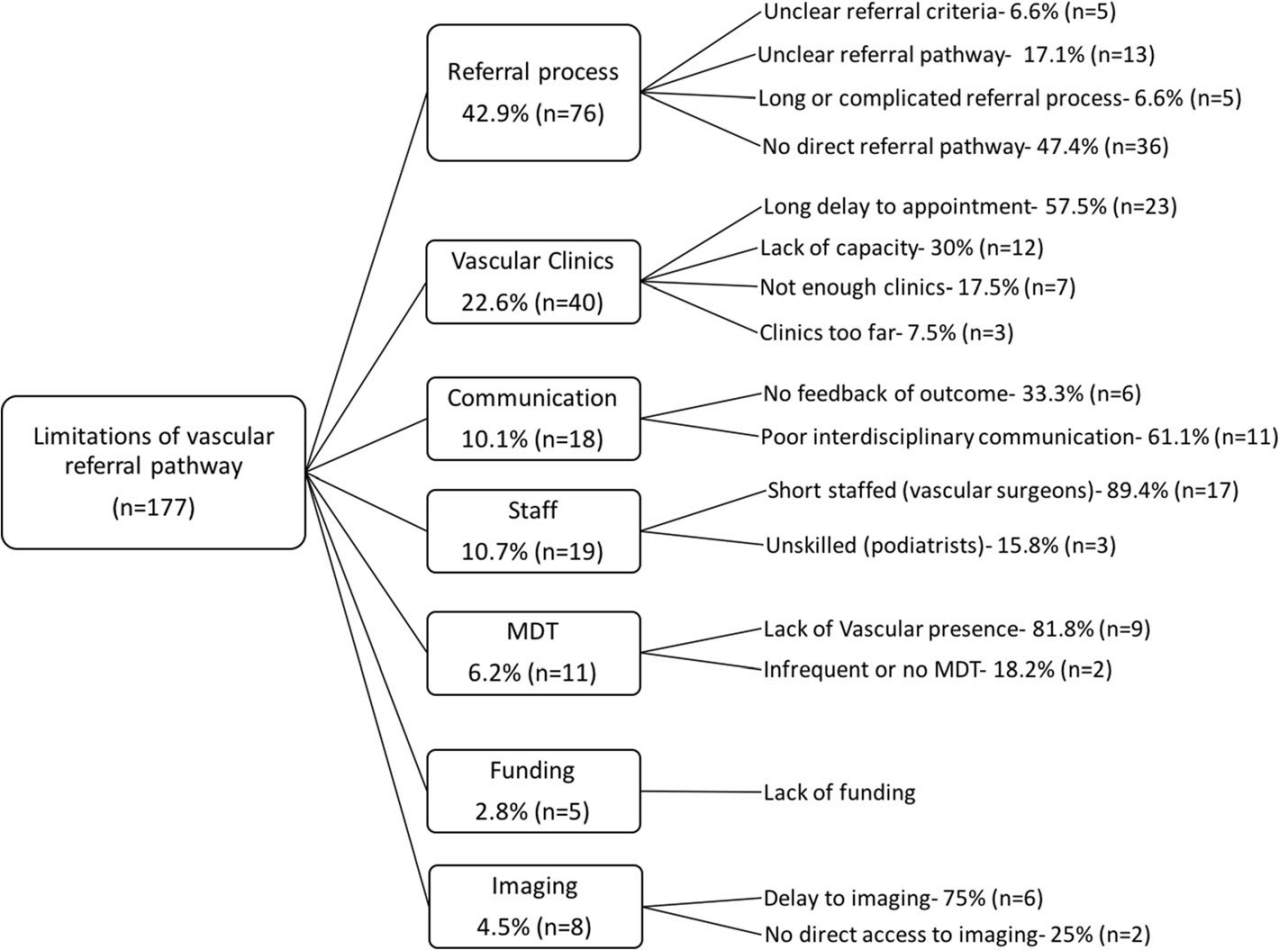
Schematic diagram of abstraction process for responses to the open question “in your opinion, what are the biggest limitations in your vascular referral pathway?”. MDFT; multidisciplinary team

In an open-ended question, we asked podiatrists what they thought the biggest limitations were in their vascular referral pathway; the abstraction process used in qualitatively analysing these responses is presented in Fig. 5 and sample quotes for each subdomain presented in Additional file 3: Table S2.

## Discussion

To our knowledge, this is the first survey to specifically evaluate national trends in PAD diagnosis in diabetes, decision-making when PAD is suspected and vascular referral pathways in detail. Although the results must be interpreted cautiously in the context of the studies limitations, they present important targets for further investigation and quality improvement. This may in turn result in improved PAD detection and a more efficient vascular referral pathway. This is becoming increasingly important with the worldwide epidemic of type 2 diabetes, which will only result in a rising prevalence of diabetic foot ulcers and a significant demand on our already strained healthcare system [20].

### Assessment for arterial disease in diabetes

Current training recommendations for podiatrists endorse history, pulse palpation with or without audible Doppler waveform assessment and ABPI as part of the standard assessment for arterial insufficiency [2]. This is supported by guidance from the International Working Group on the Diabetic Foot (IWGDF) who recommend, at minimum, a combination of history and pulse palpation in patients without active foot ulceration, and the additional use of ABPI and the assessment of arterial waveforms in those with ulceration [21]. The majority of respondents to our survey (96%) reported using a minimum combination of history and pulse palpation in PAD assessment. Although 86% reported using at least one additional non-invasive test (most commonly audible waveform assessment) very few used a complete combination of history, pulse palpation, Doppler waveform assessment and ABPI (18.8%). So although compliance with the minimum IWGDF guidance is met, complete compliance is very poor. We also found significant variation in the regions of the UK regarding what combinations of tests were used. The reason for this variation is not clear and may warrant further investigation.

Over one third of respondents were aware of missed cases of PAD in people with diabetes over the past year. Of concern was the large number of responses indicating that, in some cases, this incorrect diagnosis had contributed to a minor or major amputation. This is seldom reported in the scientific literature but there are a small number of retrospective studies which have highlighted delayed vascular referral as a cause of limb loss [11, 22]. Although the exact causes of delay are not clear from these studies, our results suggest that missed diagnosis may be a contributing factor in some cases.

Surveyed podiatrists felt confident in detecting PAD in diabetes and the majority (64%) felt they had received adequate training in PAD detection. However, a sizable proportion (23.5%) of respondents reported that they have not received adequate training in PAD detection and this may be a potential target for further investigation and quality improvement. Unsurprisingly, we found that confidence was higher in those who had a higher volume of diabetic foot practice and also those who felt they had received adequate training. It is not clear from this survey how much PAD assessment training respondents received as undergraduates. Regardless, there is also a need to maintain professional competencies after graduation and so therefore postgraduate training is also important. Our survey suggests that this may be most relevant for podiatrists with a low volume of diabetic foot practice (< 20 patients per week) and so therefore, the amount of postgraduate training may be tailored to volume of practice. Another option would be to audit the accuracy of PAD assessments locally and tailor training accordingly. In the National diabetes foot care audit (England) less than 60% of clinical commissioning groups (CCGs) could confidently say that there is training provided locally for those preforming foot checks [13]. It is not clear how much of this training is provided to podiatrists specifically and what the training entails.

Due to the confounding effects of neuropathy and arterial calcification, the detection of PAD in diabetes can be challenging and this must be also considered when discussing delayed or missed diagnosis [23].

Respondents in our survey placed high importance on history and pulse palpation in detecting PAD. Neuropathy can mask symptoms of claudication and rest pain whilst arterial calcification hinders palpation of foot pulses [24]. Pulse palpation is also limited by substantial inter-observer variation [25, 26]. High importance is also placed on audible waveform assessment using a handheld Doppler. However, this has a has a poor sensitivity (as low as 43%) in patients with diabetes as well as poor inter- and intra- observer reliability [27, 28].

IWGDF guidelines recommend the use of ABPI measurement in the assessment of the diabetic foot. They recommend considering ABPI measurements of less than 0.9 as abnormal and those between 0.9–1.3 as largely excluding PAD (GRADE recommendation: strong; Quality of evidence: low) [21]. Although, it is widely accepted that low values (< 0.9) are useful in detecting arterial disease, evidence suggests that high values (0.9–1.3 or > 1.3) cannot reliably exclude PAD in the presence of diabetes [29, 30]. This is because measurements can be falsely elevated due to the presence of incompressible calcified blood vessels [29]. Although this is acknowledged in the rationale for the IWGDF recommendation, confusingly it has still been retained in the guidance that a value between 0.9–1.3 can exclude PAD. Participants in our survey demonstrated poor knowledge of ABPI interpretation with 44.3% either falsely considering that ABPI is a reliable method to exclude PAD in diabetes or being uncertain. They also demonstrated poor knowledge of the usefulness of ABPI with only 26% of respondents routinely performing it in clinical practice. These results suggest that IWGDF guidance may need to be reviewed in order to clarify this issue.

Due to limited data and poor methodological quality of studies, there is currently little evidence to support the use of any one non-invasive diagnostic modality over another [29]. However, limited evidence suggests that TBPI and TcPO_2_ may be superior to ABPI measurements [29, 31]. In our cohort of respondents these were seldom used in clinical practice (6.2 and 0.8% respectively) and were also scored as least important in clinical decision-making. Our result suggest that there is an under utilisation of TBPI and TcPO_2_ as part of the full PAD assessment.

Unfortunately, as part of this survey we did not explore respondents technique in performing ABPI and audible Doppler waveform assessments or the cutoff thresholds for Doppler assessment.

### Vascular referral pathways

Once PAD is suspected a decision must be made as to whether it is appropriate to refer for further vascular evaluation. This is a contentious topic and a common source of confusion amongst health care professionals. The IWGDF has recommended considering further vascular imaging and revascularisation in patients with a foot ulcer and critical limb ischaemia (toe pressure < 30 mmHg, TcPO_2_ < 25 mmHg) and those with a non-healing foot ulcer and evidence of PAD (ankle pressure < 50 mmHg or ABPI < 0.5). They have also recommended to consider vascular imaging and subsequent vascular intervention, irrespective of the results of bedside tests, when an ulcer does not heal within 6-weeks despite optimal management [10, 21].

In this survey, 60% of respondents reported that they would refer any patient with suspected PAD and diabetes for further vascular assessment regardless of ulcer status. Routine referral of patients with suspected PAD but no evidence of tissue loss is not recommended in current guidelines and may increase demand on vascular services. In the presence of PAD, 16.8% of respondents reported that they would only refer non-healing ulcers after 6-weeks of conservative treatment, which could result in significant delay in management. Many respondents also reported that they would not refer for further vascular assessment prior to local debridement in cases of suspected PAD. This practice could be risky, as debridement of an ischaemic foot may fail to heal.

These results highlight a need for better collaborative working between vascular and podiatry services to clarify guidelines at a local level and ensure all healthcare providers are aware of them.

In practice, if a functional MDFT with vascular presence is established then vascular review can be arranged in a timely manner. However, in our survey over one third of respondents reported not having access to a vascular surgeon via a MDFT. This is in keeping with reports of wide variation in regional foot care services in the UK [13, 32]. We have not established what proportion of our respondents work in the community, which may influence responses to this question. Our results did however suggest that podiatrists seeing higher volume of patients were more likely to have access to a vascular surgeon via an MDFT, which suggests that this is a problem predominantly effecting lower volume clinics. It is important to note that at present there is no common agreement on what the minimum requirement is in terms of specialty presence in a MDFT and therefore it is not mandatory to have vascular presence [33]. Although universal vascular presence in the MDFT would improve access to specialist advice, analyses of open-ended responses in our survey suggest that a shortage of vascular surgeons may be a potential barrier that warrants further exploration.

When referrals have been made, almost 70% reported that their patients wait longer than 2-weeks for assessment, with the majority waiting longer than 4-weeks. Long delays, lack of capacity and too few clinics were often reported as major limitations of the vascular referral pathway. In the open responses, some podiatrists clarified that ulceration increased speed of referrals although others noted that this was not the case.

Another important finding of the present survey is the commonly perceived difficulties with the vascular referral process. There is a collective frustration with the inability to refer directly to vascular clinics, with most podiatrists having to refer via the patient’s general practitioner, which can be a further source of delay. To add to this frustration, some of those who could make direct referrals felt that the referral process was unclear or too complicated.

Surprisingly, over 70% of respondents reported that they do not receive regular feedback of outcomes from vascular consultations. This is reflective of the number who highlighted poor communication as a limitation of the vascular referral pathway. This may be because referrals are being initiated via the patient’s general practitioner who is then receiving assessment outcomes. Our results demonstrate that not all vascular referral pathways in the UK are meeting the expected standards of a ‘coordinated team approach’ to foot care. This again highlights a need for better collaborative working between vascular and podiatry services and improvement in referral pathways.

Arguably the most important step in deciding whether elective revascularisation is technically feasible or not is to obtain anatomical imaging. DUS is most often the first imaging modality of choice. Current guidelines recommend anatomical imaging whenever PAD is suspected and when it is not suspected but the ulcer has not healed despite 6-weeks of conservative treatment [21]. The Eurodiale study highlighted significant deficiencies in obtaining vascular imaging across Europe [12]. In patients diagnosed with PAD on initial vascular assessment (TcPO_2_, ABPI, TBPI), only 41% underwent anatomical imaging. In patients who did not heal after a follow up of 1-year or patients who underwent major amputation, vascular imaging was only performed in 40%. Overall, the UK had one of the lowest rates of vascular imaging when compared to other countries. In our survey, most respondents reported that they were unable to directly request a DUS and those who could were faced with long waiting times. We also found that there were differences in waiting times depending on the region of work. The reasons for this deficiency are unclear but may be related to funding, a shortage of trained staff and organisational barriers.

## Study limitations

The present study has a number of important limitations and the results should be interpreted cautiously. Firstly, although the survey was developed with multidisciplinary input its validity and reliability were not assessed.

Secondly, we have surveyed only a small proportion of podiatrists across the UK and as demonstrated there are some differences in terms of geographical spread and demographics when compared to podiatrists nationally. Furthermore, we received a relatively small number of responses from podiatrists in the private sector and it is unclear if this is representative of the overall profession or whether it’s a result of survey distribution or response bias. Additionally, not all respondents completed all of the questions in the survey, resulting in missing data. These factors also led to some difficulties in regional subgroup analysis due to small group sizes that may have resulted in a type II error.

Thirdly, responses to surveys can be very subjective and this must be considered when interpreting results. Finally, although survey studies can be powerful in highlighting what happens in clinical practice, detailed analysis is often not possible. Examples of this in the present study include the inability to ascertain the details surrounding the missed cases of PAD reported by respondents and the inability to retrospectively determine in what setting the podiatrist worked (community or hospital).

## Conclusions

This survey has highlighted poor compliance with guidelines relating to arterial disease detection in diabetes, poor utility of non-invasive tests such as (specifically ABPI, TBPI and TCO_2_) and a poor knowledge of ABPI interpretation amongst podiatrists in the UK.

We have also highlighted a lack of clarity amongst UK podiatrists regarding referral guidelines with many referring any patient with suspected PAD for further vascular assessment.

Additionaly, we have also highlighted that within the UK there is a lack of access to vascular surgeons via MDFT’s, particularly for those podiatrists seeing a lower volume of patients, lack of ability to make direct referrals for onwards vascular assessment, lack of feedback regarding vascular assessment outcome and long waiting times for vascular review and DUS imaging.

The above findings are important targets for further investigation and quality improvement.

## Additional files

Additional file 1: **Figure S1.** UK National podiatry survey. (PDF 253 kb)

Additional file 2: **Table S1.** Geographical distribution of podiatrists in the United Kingdom compared to the geographical distribution of respondents to our survey. (DOCX 51 kb)

Additional file 3: **Table S2.** Sample quotes from answers to the open question “in your opinion, what are the biggest limitations in your vascular referral pathway?”. (DOCX 141 kb)

## Abbreviations

ABPI: Ankle brachial pressure index
CCG: Clinical commissioning group
DUS: Duplex ultrasound
FPT: Foot Protection Team
IWGDF: International working group on the diabetic foot
MDFT: Multidisciplinary Foot Care Team
NHS: National Health Service
NICE: National Institute of Health and Care Excellence
PAD: peripheral arterial disease
TBPI: Toe brachial pressure index
TcPO_2_: Transcutaneous pressure of oxygen
UK: United Kingdom
