# Supplementary material for: Management of peripheral arterial disease in diabetes: a national survey of podiatry practice in the United Kingdom
Source: J Foot Ankle Res. 2018 Jun 8;11:29. doi: 10.1186/s13047-018-0270-5 (PMC5994074; doi:10.1186/s13047-018-0270-5)
Supplement: Supplementary file 1 — Figure S1. UK National podiatry survey. (PDF 253 kb) [file 13047_2018_270_MOESM1_ESM.pdf]

Thank you for participating in our survey

Dear Colleague,

Thank you for taking the time participate in this survey. Diabetic foot complications are frequent and extremely challenging to manage. Peripheral arterial disease (PAD) is a major risk factor for the development of foot ulceration, limb loss and overall mortality. However, its detection in diabetes can be difficult.

In this National survey, we aim to explore:

1. current practice patterns in PAD screening, and

2. Vascular referral pathways

With your help we hope to identify areas for potential quality improvement in the future. The Survey should take approximately 5 minutes to complete. With your help we hope to identify areas for potential quality improvement in the future.

Thank you for participating!

Imperial College Healthcare NHS
NHS Trust

Peripheral Arterial Disease in Diabetes; A National Survey of Podiatry Practice

Demographics

* What is your occupation?

* How many years have you worked in this capacity?

* In which sector do you work?

- NHS
- Private

If you work in the NHS, what is your banding?

* In which region of the UK do you work?

- East
- East Midlands
- London
- North East
- North West
- Northern Ireland
- Scotland
- South East
- South West
- Wales
- West Midlands
- Yorkshire And The Humber

* How many diabetic patients do you see per week?

* How long is your typical appointment slot with each patient?

* Do you have access to advice from a vascular surgeon via an MDT?

- Yes
- No
- Unsure

Peripheral Arterial Disease in Diabetes; A National Survey of Podiatry Practice

Screening for arterial disease in diabetic patients

* Which patients with diabetes do you routinely screen for peripheral arterial disease (PAD)?

- All patients
- Neuropathy
- Ulceration
- Foot infection
- Charcot arthropathy
- Gangrene

* How confident are you in your ability to accurately screen for PAD in diabetic patients?

0 (not at all confident) 50 (unsure) 100 (very confident)

* Do you feel that you have received adequate training in detecting PAD?

- Yes
- No
- Usnure

* Which of the following do you routinely perform as part of your screening (tick all that apply) for PAD?

- [ ] History
- [ ] Capillary refill time
- [ ] Palpation of foot pulses
- [ ] Audible handheld Doppler waveform assessment
- [ ] Ankle-brachial pressure index (ABPI)
- [ ] Toe-brachial pressure index (TBI)
- [ ] Transcutaneous oxygen tension (TcPO2)

* Please score the following options to indicate their importance to your clinical assessment.

	N/A (not used in clinical assessment)	Not at all important	Not important	Unsure	Important	Very important
History	○	○	○	○	○	○
Capillary refill time	○	○	○	○	○	○
Palpation of foot pulses	○	○	○	○	○	○
Audible handheld Doppler waveform assessment	○	○	○	○	○	○
Ankle- brachial pressure index (ABPI)	○	○	○	○	○	○
Toe-brachial pressure index (TBI)	○	○	○	○	○	○
Transcutaneous oxygen tension (TcPO2)	○	○	○	○	○	○

* Do you think ABPI (<0.9 considered abnormal) is a reliable test to exclude PAD in diabetic patients?

○ Yes

○ No

○ Unsure

* Approximately how many patients do you typically refer for further vascular assessment?

Per month __________

Per year __________

* If you suspect PAD in a patient with diabetes, when would you refer for further Vascular assessment?

- [ ] If there is any active foot ulceration
- [ ] If there is an ulcer which has not improved within 6 weeks despite optimal management
- [ ] If there is evidence of infection
- [ ] If they need local debridement
- [ ] I would refer any patient with suspected PAD and diabetes for further assessment

* When you do refer a patient for further Vascular assessment, do you always receive the outcome of the patients visit?

- ○ Yes
- ○ No

* In the last one year, are you aware of any patients who had PAD which was previously missed on clinical examination?

- ○ Yes
- ○ No
- ○ Unsure

If so, did this contribute to a minor/ major amputation in any of these cases?

- ○ Yes
- ○ No
- ○ Unsure

Peripheral Arterial Disease in Diabetes; A National Survey of Podiatry Practice

Vascular referral pathway

* Can you directly refer a patient for an arterial duplex ultrasound scan ?

- ○ Yes
- ○ No
- ○ Unsure

If yes, how long does it take to have a duplex ultrasound scan performed?

- <1 week
- 1-2 weeks
- 2-3 weeks
- 3-4 weeks
- >4 weeks

If yes, is the waiting time clinically appropriate?

- Appropriate
- Too Long
- Unsure

* On average how long do your patients have to wait for a Vascular assessment by a Vascular Surgeon?

- <1 week
- 1-2 weeks
- 2-3 weeks
- 3-4 weeks
- >4 weeks

* Do you think this is an appropriate waiting time?

- Appropriate
- Too long
- Unsure

In your opinion, what are the biggest limitations in your Vascular referral pathway?

* In the past one year, of the patients you referred for Vascular assessment, what proportion required Vascular intervention?

- <10%
- 10-25%
- 25-50%
- 50-75%
- >75%

Peripheral Arterial Disease in Diabetes; A National Survey of Podiatry Practice

Thank you for taking the time to complete this survey!

Imperial College Healthcare NHS
NHS Trust
