# Supplementary material for: Management of peripheral arterial disease in diabetes: a national survey of podiatry practice in the United Kingdom
Source: J Foot Ankle Res. 2018 Jun 8;11:29. doi: 10.1186/s13047-018-0270-5 (PMC5994074; doi:10.1186/s13047-018-0270-5)
Supplement: Supplementary file 2 — Table S1. Geographical distribution of podiatrists in the United Kingdom compared to the geographical distribution of respondents to our survey. (DOCX 51 kb) [file 13047_2018_270_MOESM2_ESM.docx]

|  | **National statistics (%)** | **Survey respondents (%)** |
| --- | --- | --- |
| **East midlands** | 5.9 | 3.9 |
| **East** | 4.7 | 2.8 |
| **London** | 9.6 | 13.8 |
| **North East** | 5.7 | 1.1 |
| **North West** | 15.2 | 14.8 |
| **Northern Ireland** | 5.6 | 1.4 |
| **Scotland** | 17.0 | 15.9 |
| **South East** | 6.7 | 18.0 |
| **South West** | 7.2 | 7.0 |
| **Wales** | 6.7 | 8.1 |
| **West Midlands** | 8.1 | 2.8 |
| **Yorkshire and the Humber** | 7.7 | 9.9 |
