# Supplementary material for: Management of peripheral arterial disease in diabetes: a national survey of podiatry practice in the United Kingdom
Source: J Foot Ankle Res. 2018 Jun 8;11:29. doi: 10.1186/s13047-018-0270-5 (PMC5994074; doi:10.1186/s13047-018-0270-5)
Supplement: Supplementary file 3 — Table S2. Sample quotes from answers to the open question “in your opinion, what are the biggest limitations in your vascular referral pathway?”. (DOCX 141 kb) [file 13047_2018_270_MOESM3_ESM.docx]

| **Category** | **Sub-category** | **Sample quote** |
| --- | --- | --- |
| Referral process | Unclear referral criteria | “I'm not aware of the criteria of referral to vascular.”  “We have no referral guidelines so it's unclear who to send and when.” |
|  | Unclear referral pathway | “Being unaware of correct mechanisms of referral.”  “Informal pathway.”  “Lack of knowledge of the pathways.”  “Ways of communication between departments are unclear and changing often. It is so confusing sometimes.” |
|  | Long or complicated referral process | “Too much red tape!!”  “Lengthy referral.” |
|  | No direct referral | “We cannot directly refer our patients we have to write to the GP to request a referral, we can refer suspected CLI patients as a matter of urgency via faxing a letter to the secretaries of the consultant.”  “No formal referral pathway established, referral through GP.”  “Having to refer to a GP first. This wastes a lot of time.” |
| Vascular clinics | Long delay to appointment | “Length of time it takes for the patient to be seen by vascular consultant.”  “Time taken to be seen when referred as urgent.”  “Ulceration increases speed of referral, thus if no current ulceration, waiting time is significantly increased.” |
|  | Lack of capacity | “Limited vascular clinic as we are now a hub.”  “Capacity of specialist vascular consultants clinics”  “The outpatient clinics are over booked and the staff have to fight to get true CLIs seen in a timely way.” |
|  | Not enough clinics | “Amount of vascular consultant out patient appointments in our spoke hospital.”  “Availability of outpatient clinics in the community hospitals I work in.” |
|  | Clinics too far | “The patient and their relatives are reluctant to put the time and effort into getting to regional centres of vascular expertise.”  “Vascular specialist based at hub hospital, 20 miles from our foot clinic.” |
| Communication | No feedback of outcome | “We rarely get outcome sent back we have to rely on what the patient reports happened at vascular.”  “We refer, but all feedback to GP.”  “Communication, I very seldom get information back from the vascular department unless it is in our joint vascular clinic as part of the MDT.” |
|  | Poor interdisciplinary communication | “When Surgeon is away and we are not informed there will be no Vascular in attendance in the clinic.”  “Correspondence often not read or lost.”  “Not being taken seriously GPs not considering my diagnosis.” |
| Staff | Short staffed (vascular surgeons) | “…it is lack of Vascular surgeons that delays/limits pathway.”  “Lack of vascular staff.”  “Lack of a vascular nurse.”  “Half a day clinic once a month for the vascular surgeon is not enough clinic time” |
|  | Unskilled (podiatrists) | “There are clinics staffed by staff with sub optimal skills- hence the missed PAD and subsequent amputations.”  “More training and confidence of front line pad assessors needed.” |
| MDT | Lack of vascular presence | “It would be useful if the diabetic foot MDT had a vascular input within that team.”  “Lack of integration from vascular team. Vascular not willing/able to be involved in MDT clinic.”  “No direct vascular involvement in MDT.” |
|  | Infrequent or no MDT | “The MDT is quite ad hoc.” |
| Funding | Lack of funding | “Management emphasise on the importance of saving on costs and therefore this can hinder referrals.”  “There is absolutely no funding for investment in this despite it's growing importance in our ageing caseload & increase in foot ulceration referrals to the service.” |
| Imaging | Delay to imaging | “Delays in arterial duplex scanning.”  “Too long a wait for imaging.” |
|  | No direct access to imaging | “Access to Duplex scans.”  “Getting the duplex scan.” |
